# Caught in the act: phenotypic consequences of a recent shift in feeding strategy of the shark barnacle *Anelasma squalicola* (Lovén, 1844)

**DOI:** 10.1007/s00435-015-0296-1

**Published:** 2016-01-08

**Authors:** Anders Ommundsen, Christoph Noever, Henrik Glenner

**Affiliations:** Department of Biology, University of Bergen, Bergen, Norway; CMEC, Natural History Museum, University of Copenhagen, Copenhagen, Denmark

**Keywords:** Evolutionary transition, Feeding strategy, Parasitism, Selection, Macro-evolution

## Abstract

*Anelasma squalicola* is a barnacle found attached to deep-water lantern sharks of the family Etmopteridae and is the only known cirriped on fish hosts. While *A. squalicola* is equipped with mouth and thoracic appendages (cirri), which are used for suspension feeding in conventional barnacles, its attachment device (peduncle) appears to have evolved into a feeding device, embedded into the tissue of its host. Here we demonstrate, through comparisons of the feeding apparatuses between *A. squalicola* and conventional suspension-feeding barnacles, that mouthparts and cirri of *A. squalicola* are highly reduced, and incapable of suspension-feeding activities. We show that in conventional suspension-feeding barnacles strong symmetries exist within these vital trophic structures. In *A. squalicola* strong asymmetries are widespread, indicating that those structures have been uncoupled from natural selection. The digestive tract is consistently empty, suggesting that feeding via cirri does not occur in *A. squalicola*. In addition, comparisons of stable isotope ratios (δ^13^C and δ^15^N) between *A. squalicola*, its shark host, and a conventional suspending feeding barnacle indicate that *A. squalicola* is taking nutrition directly from its host shark and not from the surrounding water. Our results strongly indicate that this barnacle has abandoned suspension feeding and now solely relies on obtaining nutrition from its host by a de novo evolved feeding mechanism.

## Introduction

**Fig. 1:**
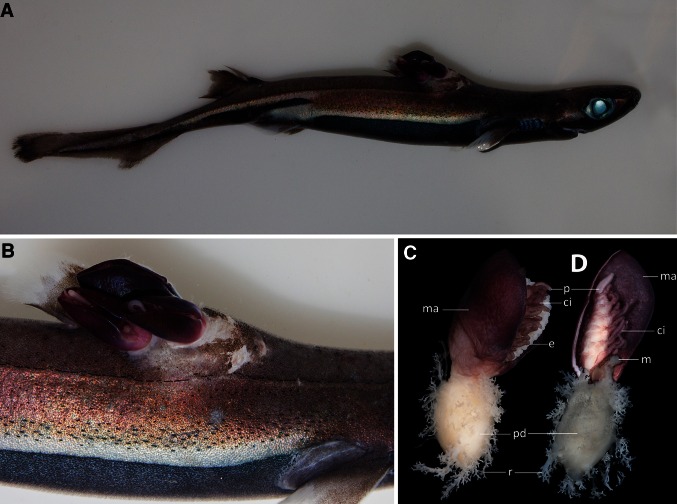
**a** The velvet belly lantern shark, *E. spinax*, with three specimens of *A. squalicola* embedded at the base of the first dorsal fin. **b** Detailed view of the *A*. *squalicola* specimens shown in (**a**). **c** *A*. *squalicola* habitus showing the capitulum and the exposed peduncle. The *whitish lower half* constitutes the modified peduncle, dissected free from the tissue of its shark host. **d** *A*. *squalicola* with the part of the mantle facing the viewer removed. *ci* cirri, *e* egg mass, *m* mouth, *ma* mantle, *p* penis, *r* rootlets

Thoracican barnacles are a group of marine crustaceans that share a suite of unique morphological characters adapted to their adult lifestyle as permanently attached, suspension-feeding organisms. The ancestral body form within the group consists of a fleshy stalk-like device, the peduncle, which attaches to a substrate, and erects the rest of the body, the capitulum, from it (Darwin 1851). The capitulum is covered by a mantle that surrounds the body and feeding appendages. In most thoracican barnacles, calcareous plates are embedded in the mantle for protection. In some species, including *Anelasma* squalicola (Lovén, 1844), these plates are reduced in size or entirely missing. In contrast to other stalked barnacles, that use the peduncle to elevate the capitulum above the substrate, *A. squalicola* has embedded this device into the tissue of its hosts (Fig. 1), various deep-water sharks of the family Etmopteridae (Long and Waggoner 1993; Yano and Musick 2000). Despite *A. squalicola*’s intriguing and highly aberrant morphology this barnacle has rarely been studied. It first appeared in the scientific literature in a study from the eighteenth century conducted by the Norwegian naturalist Gunnerus (1763). Gunnerus was studying the velvet belly lantern shark, *Etmopterus* spinax (Linnaeus, 1758), when he came across a specimen parasitized by what would later be named *A. squalicola*. He published his finding and correctly identified the specimen as a crustacean. However, Gunnerus’ attention was on the shark and not on the attached barnacle specimen, so he neither thoroughly described it nor gave it a name. Due to the obscure journal in which Gunnerus’ paper was published, his description remained largely overlooked for decades (Broch 1919). Nearly a century later, the Swedish zoologist Lovén (1844) identified the shark-attached crustacean as a barnacle and described it as *Alepas squalicola*. When Charles Darwin investigated the species, he realized that it was assigned to the wrong genus and re-described the species in his monograph on Cirripedia (Darwin 1851). In this outstanding contribution, he ascribed it to a new monotypic genus, *Anelasma* Darwin 1851. Among other unusual morphological structures, Darwin found it remarkable that the peduncle of the specimen, instead of elevating the capitulum from the site of attachment, had penetrated the skin of the shark and deeply embedded a globular device into the muscle tissue of the host. Darwin homologized the stalk of *A. squalicola* with the peduncle of lepadomorph barnacles (synonymous with Lepadidae, which, along with Balanidae and Veruccucidae comprised the Cirripedia at the time of Darwin’s monograph). He observed that the external part of the embedded peduncle was equipped with small branching rootlets, which apparently penetrated into the muscle tissue of the shark. Despite the fact that Darwin did not find food remains in the stomach of the dissected specimen, it did not occur to him at this point that the animal’s nutritional requirements might be fulfilled by means other than the alimentary tract, namely via the embedded peduncle. Instead he recapitulated his investigation of the feeding mode of *A. squalicola*: “As the whole of the peduncle is imbedded, and as the mouth is probosciformed, with the labrum a little curled over the adductor muscle, I conclude that this Cirripede can reach minute animals crawling by on the surface of the shark’s body” (Darwin 1851). The possible parasitic nature of *Anelasma* became obvious to him when he became aware of the rhizocephalan barnacles through the pioneer work of the German zoologist Müller (1862), but he never published his thoughts on this matter (Burkhardt 1996). Although the parasitic lifestyle is considered likely, it is unclear whether the barnacle is a true parasite obtaining nutrition solely from its host or if it is still capable of suspension feeding. Smith (1906) interpreted the rootlets of the peduncle as a trophic structure, and Broch’s (1919) conclusion was that *A. squalicola* is, if not wholly at least partly, feeding on the shark host via its peduncle. Yano and Musick (2000) showed that *A. squalicola* has a negative impact on the reproductive organs of its host, causing those authors to suggest that *A. squalicola* acts as a parasite and directly removes nutrients from the shark. However, an alternative explanation is that the host itself redirects resources from the developing reproductive organs to the immune system, in order to fight and get rid of the non-parasitic, but tissue-embedded barnacle. This is not an unreasonable hypothesis because parasitism, unlike epibiosis, is uncommon among cirripedes and has only evolved a few times. One instance is the cirripedian suborder Rhizocephala, all of which have crustacean hosts (Høeg 1995). Within Thoracica the only other taxon with morphological reductions and a host-tissue-embedded peduncle comparable to that of *A. squalicola* is the polychaete-infesting *Rhizolepas* Day, 1939, with two species recognized (Day 1939; Zevina 1968). This makes *A. squalicola* the only known barnacle with a fish as a required host. Although most studies have interpreted *A. squalicola* as a parasite, the documentation is weak and it is not clear whether *A. squalicola*, in addition to potentially parasitizing the shark, remains capable of suspension feeding. If this is not the case, then the cirri, mouthparts, and most likely also the alimentary tract have lost their function, meaning that these structures either are under selection for new functions, or not under selection at all. The present study addresses these questions.

## Materials and methods

### Sampling

Fifty-three specimens of the velvet belly lantern shark, *Etmopterus spinax* (Fig. 1a) parasitized by *A. squalicola* were collected in the Sognefjord, western Norway, in November 2012 and May 2013. The sharks were caught between 200 and 250 m depths using a bottom trawl. Most *A. squalicola* were dissected out from the shark’s tissue on board, and some were fixed embedded in pieces of muscle tissue for later dissection. The specimens were preserved in 4 % formalin and transferred to 70 % ethanol for storage. One specimen of *A. squalicola* was collected from the Raunefjord, south of Bergen, Norway using longlines and preserved in 4 % formalin. Two specimens of *A. squalicola* were collected in the Azores Archipelago in 2008, from the great lanternshark, *Etmopterus princeps* Collett, 1904 using longlines.

Specimens of the acorn barnacle *Balanus balanus* (Linnaeus, 1758) were collected in the subtidal in the vicinity of Bergen, Norway, at a depth of 5–15 m. *Balanus balanus* specimens were preserved in 70 % ethanol after tissue samples were taken for isotopic analyses. Specimens of the pedunculate barnacle *Lepas anatifera* Linnaeus, 1758 were sampled in the Azores Archipelago in 2008, and preserved in 70 % ethanol.

### Morphology of the mouthparts

From the three barnacle species, *A. squalicola*, *B. balanus*, and *L. anatifera*, the oral cones of one specimen of each, and the mandibles of seven *A. squalicola* and six of each of the conventional barnacles were dissected out under a dissection microscope. They were dehydrated in an ethanol series and critical point dried in CO_2_ using a Balzers CPD 030. The dried oral cones and mandibles were mounted on SEM stubs with conducting carbon tape and sputter coated with gold/palladium using a BIO-RAD E5400 SEM coating system. Observations and photographs were made with a FEI Quanta FEG 450 scanning electron microscope operated at 10 kV. Adobe Photoshop CS5 was used to assemble the figures.

### Thoracic appendages

The cirri of 100 specimens of *A. squalicola* were checked for abnormalities under a dissection microscope. Examples of cirri abnormalities were photographed using a stereomicroscope with a Nikon Digital Sight DS-U1 camera. Adobe Photoshop CS5 was used to assemble the figures. In order to investigate the functionality and morphology of the mouth appendages in *A. squalicola*, they were compared to those of a pedunculate and an acorn barnacle, *L. anatifera* and *B. balanus*, respectively. Most emphasis was put on the mandibles, but the general morphology of the oral cones was also examined.

### Isotopic (δ^13^C and δ^15^N) analyses preparation

Tissue samples from 15 specimens of the host shark (*E. spinax*) and of *A. squalicola* were collected on board, prior to preservation of the specimens, and immediately stored at −20 °C. From *E. spinax*, the tissue samples were taken from the white muscle tissue on the dorsal side behind the head. From *A. squalicola*, the tissue was sampled from the soft mantle. From five specimens of *B. balanus,* muscle tissue was retrieved from freshly killed specimens in the laboratory and immediately frozen at −20 °C. Without prior thawing, the tissue samples were freeze-dried using a CHRIST Alpha 1-2 LDplus freeze dryer. Dried samples were ground to a fine powder using a mortar and pestle. Each sample was weighed to 1 mg (±0.2 mg), and encapsulated in tin capsules (5 × 9 mm). Samples were treated according to the encapsulation procedure provided by the Stable Isotope Facility at the University of California, where the analysis was carried out using Elemental Analysis—Isotope Ration Mass Spectrometry (EA-IRMS).

### Digestive tract

The digestive tract (pharynx, stomach, and intestine) of 30 specimens of *A. squalicola* that had been immediately fixed in formalin after capture were dissected under a dissection microscope and screened for food particles.

## Results

### Mouth appendages

The mouth appendages (trophi) consist of the same structures in all thoracican barnacles. This includes a labrum with its associated mandibular palps, mandibles, maxillules and maxillae (Høeg et al. 1994). These structures are highly reduced in *A. squalicola*. Previous studies have therefore created some confusion with respect to the terminology of these appendages.

#### Oral cones

**Fig. 2:**
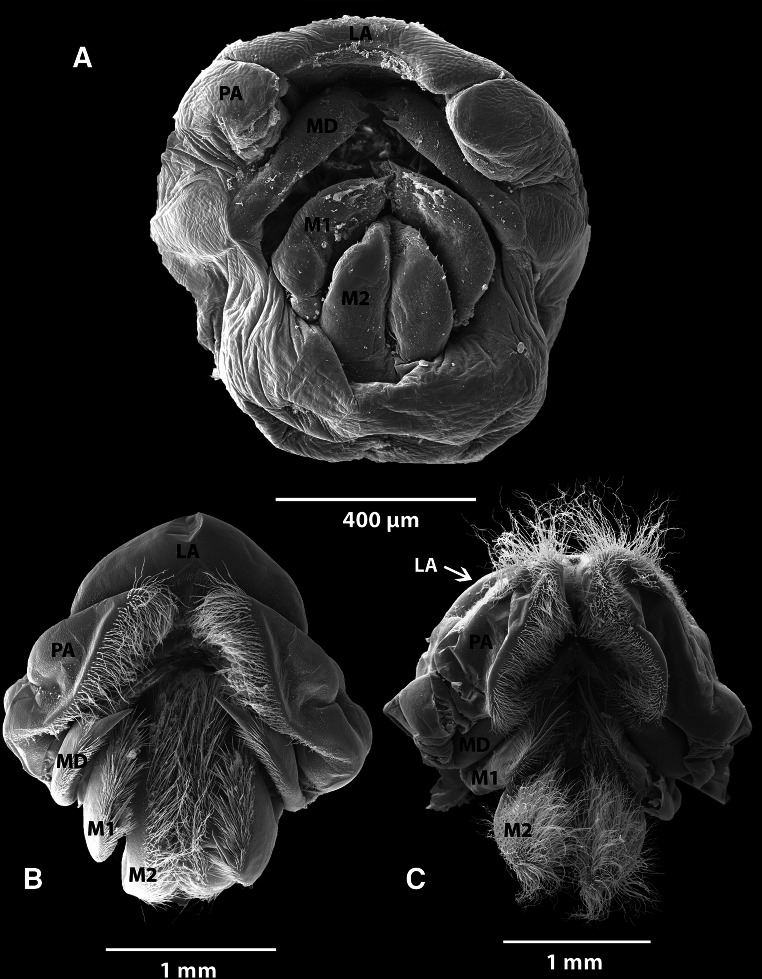
SEM photographs of the oral cones with mouth appendages of the three barnacle species. **a** *Anelasma squalicola*, **b** *Lepas anatifera*, **c** *Balanus balanus*. *LA* labrum, *PA* mandibular palp, *MD* mandible, *M1* maxillule, *M2* maxilla

Oral cones of all three species are presented in Fig. 2. The mouth appendages of *A. squalicola* (Fig. 2a) are evidently reduced in contrast to the two conventional barnacle species and are almost without pronounced denticles or spines. The mandibles of some *A. squalicola* specimens possess areas with diminutive denticles, which might be homologous to the denticles found on other barnacle species. Even though the labrum of *L. anatifera* and *B. balanus* is partly or wholly covered by the mandibular palps in the oral cones shown here, examination showed that all mouth appendages, including the labrum, of these two species possess a well-developed setation. The size of *A. squalicola*’s mandibular palps is also remarkably small, and they do not cover the labrum. In *A. squalicola*, the paired maxillules, which are just slightly smaller and situated below the mandibles, possess a spinose edge. Below the maxillules is a pair of blunt, almost smooth maxillae, possessing only few minute spines. These maxillae have the same appearance as the small mandibular palps, also clearly rudimentary compared to those of *L. anatifera* and *B. balanus*.

#### Mandibles

##### *Anelasma squalicola*

**Fig. 3:**
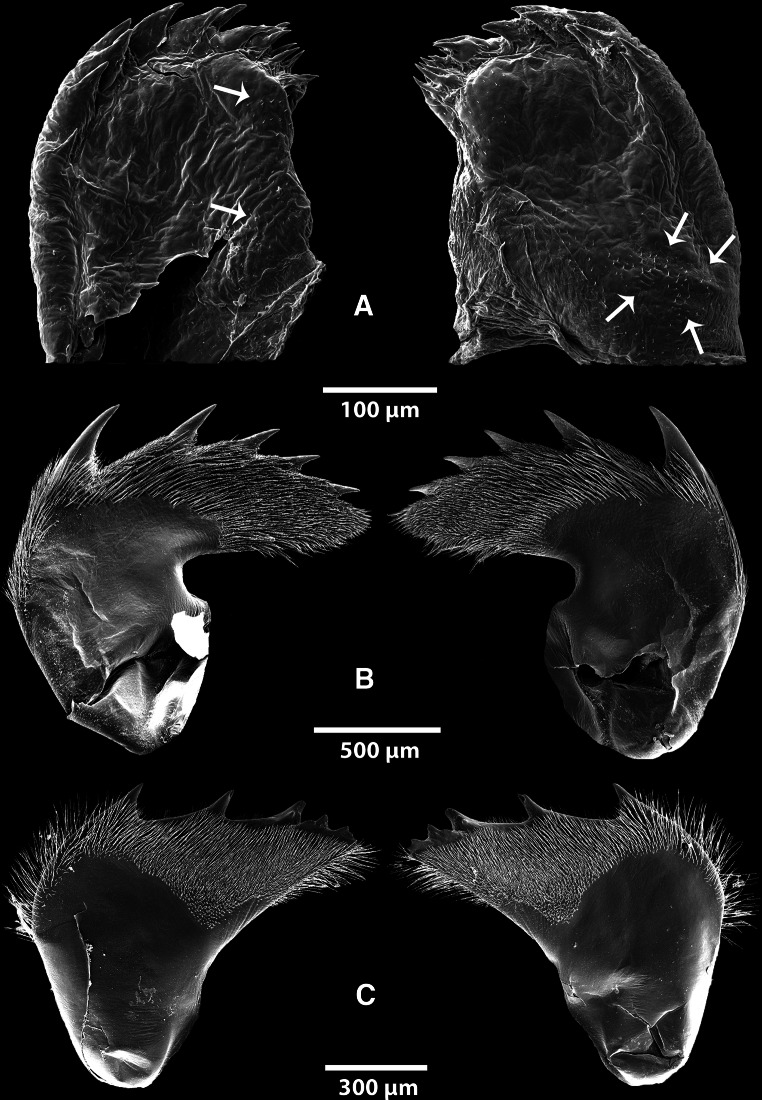
**a** Mandibles from *A. squalicola*, spines resembling rudimentary setae or denticles are indicated with *white arrows*. **b** Mandibles from *L. anatifera*, **c** mandibles from *B. balanus*. Note the symmetrical organization of setae and denticles in (**b**) and (**c**)

**Fig. 4:**
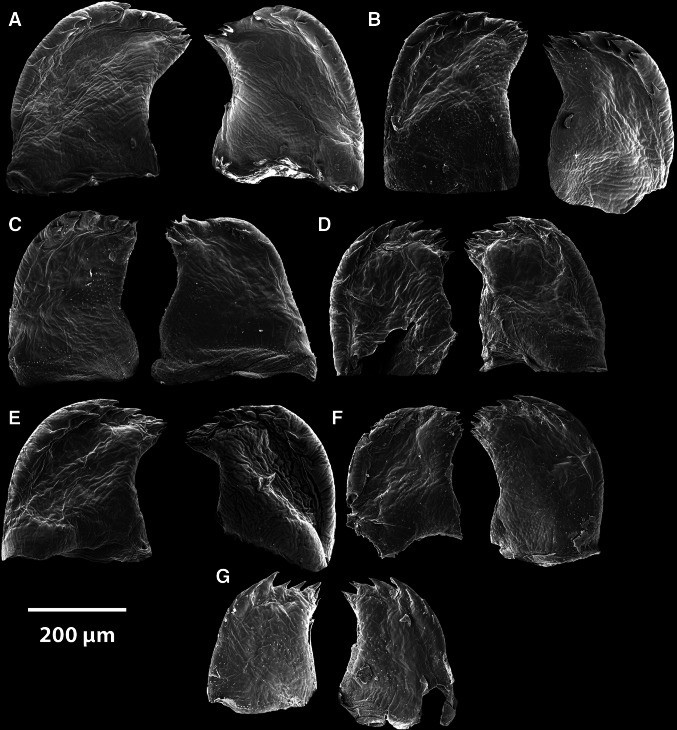
Mandible pairs from seven specimens of *A. squalicola*

All mandibles are remarkably small and fragile, compared with those from specimens of *L. anatifera* and *B. balanus* (Fig. 3). In contrast to those in other barnacles, the mandibles of *A. squalicola* demonstrate no consistent patterns concerning the tooth-covered distal edges (Fig. 4). The distribution of teeth at the distal edges of the mandibles within each individual shows strong left–right asymmetries. The morphology and number of the teeth is highly variable. The teeth alternate irregularly between being paired (bifid) or single and in size. Due to variations between specimens and individual left–right asymmetries, a generalized morphology of the *A. squalicola* mandibles can hardly be provided (Fig. 4). The only consistent similarity of the mandibles is the clustering of smaller teeth toward the proximal part of the median mandible edge facing the esophagus. This part of the mandible (clustered with smaller teeth) could be interpreted as the molar. The more distally positioned region with the generally larger teeth would then represent the incisor process. There is, however, no sharp distinction between these two regions in *A. squalicola*. The number of incisor teeth (with possible bifid teeth counted as two separate teeth) ranges from about five to ten per mandible, while the clustered region consists of four to ten smaller teeth. Only in one specimen (Fig. 4g) is the number of teeth the same in both the left and right mandibles. The specimen showing the highest variation between left and right mandibles (Fig. 4a) has a left mandible with ten incisor teeth and seven clustered region teeth, while the right mandible has five incisor teeth and five clustered region teeth.

##### *Lepas anatifera*

**Fig. 5:**
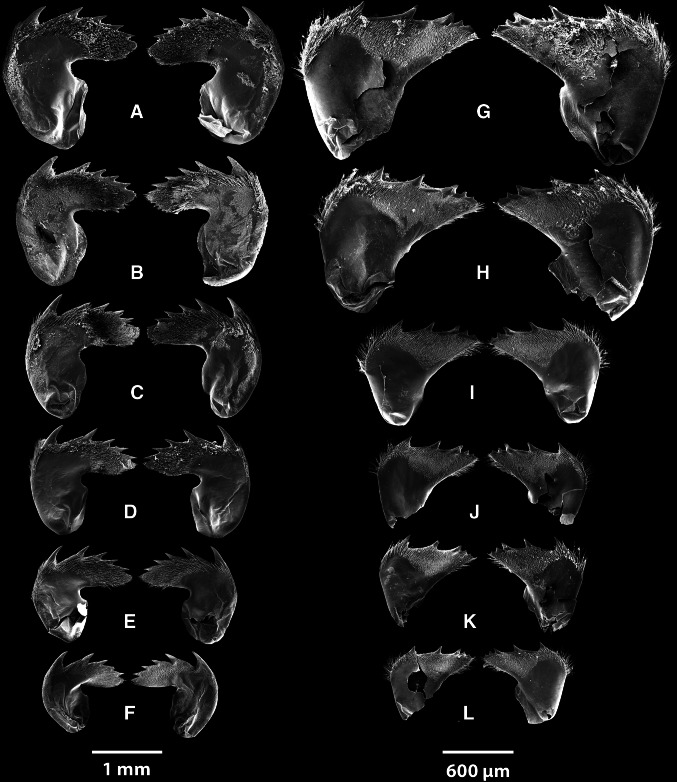
SEM photographs of mandible pairs from the two conventional suspending feeding barnacle species. **a**–**f** Six specimens of *L. anatifera.* **c** Left mandible showing an abnormal tooth pattern, with the second, third and fourth teeth being bifid. **g**–**l** Mandible pairs from six specimens of *B. balanus*

The mandibles of *L. anatifera* (Fig. 5a–f) show clear symmetries in the organization of teeth along the distal edge, which carries five long incisor teeth that gradually decrease in size toward the edge facing the esophagus. A sixth molar tooth, totally covered in denticles, is situated innermost on the distal edge. The shape of the mandibles is consistent during growth of the species. As Fig. 5 indicates, a size range of specimens was investigated. The left mandible of one pair was found to be an exception to this pattern, as its incisor teeth 2, 4 and 5 were bifid (Fig. 5c). All six pairs have the same symmetrical organization of setation and denticles (Fig. 5b) and are very robust.

##### *Balanus balanus*

The mandibles of *B. balanus* (Fig. 5g–l) show clear symmetries concerning the tooth-covered distal edge. It carries three incisor teeth, the second of which is bifid, while the third is partly bifid. The fourth and innermost tooth is a molar process. As in *L. anatifera*, the teeth gradually decrease in size toward the edge facing the esophagus. The shape of the mandibles shows a consistent and nearly identical pattern between the examined specimens, and it is obvious that the size of the mandible increases proportional with overall size of the individuals. All six pairs of examined mandibles have the same symmetrical organization and are very robust.

### Thoracic appendages

**Fig. 6:**
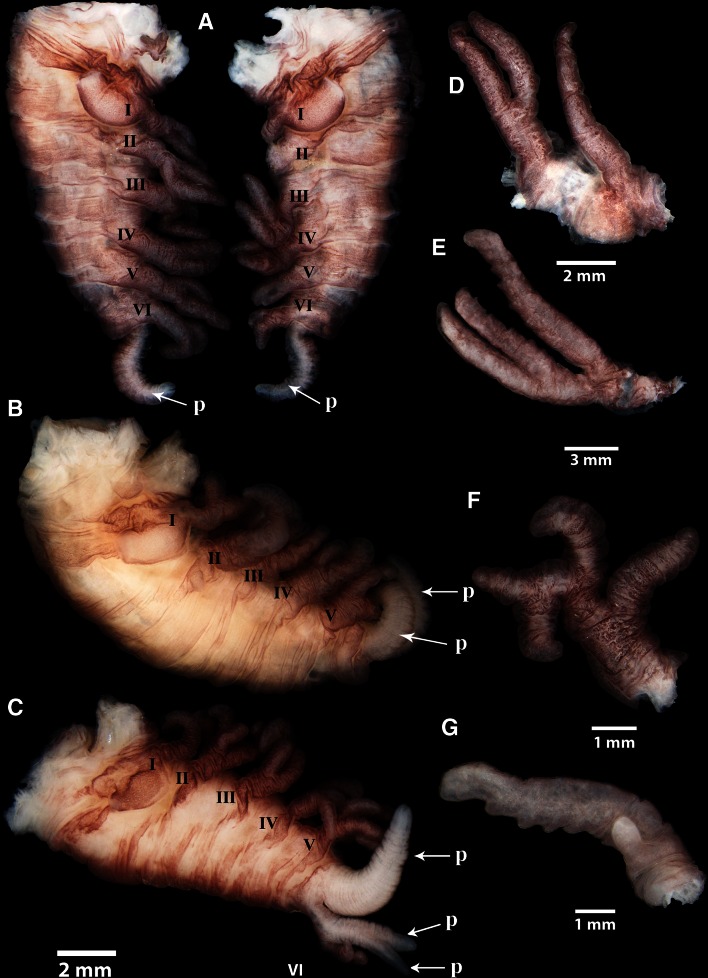
Light microscopy images of abnormally shaped cirri in *A. squalicola*. **a**–**c** Lateral view of three specimens dissected free from peduncle and mantle. **a** The specimen has been cut in two in a sagittal plane. Right half is lacking cirri I and II, and cirrus V is biramous but strongly reduced. Left half is normal. **b** The specimen is lacking a whole pair of cirri and has an additional penis. **c** The specimen has one larger and two additional smaller penises. Sixth cirri pair consists of only one cirrus, which is located dorsally to the two additional penises. **d** Uniramous and “normal” biramous cirri. **e** Triramous cirrus, with traces of segmentation in the two upper branches in figure. **f** Tetraramous cirri. **g** Very unequal branches on cirrus

Thoracican barnacle have six pairs of segmented, biramous thoracic appendages (cirri), which are part of the feeding apparatus in suspension-feeding barnacles, and a penis located dorsally to the sixth cirri pair. The cirri of 100 specimens of *A. squalicola* from Norway were investigated to examine their functional morphology. All examined specimens had reduced cirri, as they were completely devoid of setation (Fig. 6) and traces of annulations were only observed in a few specimens. The majority of the specimens (56 %) had abnormal cirri patterns, as they were partially lacking, had abnormal shaped cirri or were left–right asymmetrical.

“Description of abnormalities” section describes the abnormalities and asymmetries observed. Examples of the abnormalities are shown in Fig. 6.

#### Description of abnormalities

##### *Uniramous*:

cirrus is lacking a second branch as seen in normal biramous cirri (Fig. 6d).

##### *Triramous*:

cirrus has an extra branch, branching into three instead of two (Fig. 6e).

##### *Tetraramous*:

cirrus has two extra branches, branching into four instead of two (Fig. 6f).

##### *Very unequal branches*:

two branches on one cirrus are of very unequal length; often one branch is <1/3 of the other (Fig. 6g).

##### *Lacking two different cirri*:

two cirri from different pairs are lacking (Fig. 6a).

##### *Lacking pair*:

a whole pair of cirri is lacking, leaving fewer than the typical six pairs of cirri (Fig. 6b).

##### *Lacking cirrus*:

one of two cirri in a pair is lacking (Fig. 6a, c).

##### *Other*:

more than one penis (Fig. 6b, c), sixth pair of cirri located behind penis (Fig. 6c), or reduced biramous cirrus (Fig. 6a).

56 % of the *A. squalicola* specimens from the Sognefjord had one or several abnormally formed cirri (Table 1). The degree of abnormalities varied, as some specimens displayed several different types of abnormalities, while others had only one type. 38 % of the examined specimens had one type of abnormality, while one specimen had four different types of abnormalities. The individual with the most abnormalities had a cirrus that was tetraramous and, in addition, was lacking two whole pairs of cirri and one cirrus from a third pair. Specimens found to have only one abnormality could have several cases of this abnormality. For example, one specimen had nine uniramous cirri of a total of twelve cirri (six pairs).

**Table 1:** The percentage of individuals of *A. squalicola* within the studied population with zero to multiple cirri asymmetries

Asymmetries or abnormalities/specimen	%
0	44 specimen—44 %
1	38 specimen—38 %
2	13 specimen—13 %
3	4 specimen—4 %
4	1 specimen—1 %

In total 100 specimens were examined

The thoracic appendages of the three additional specimens of *A. squalicola*, supplied from earlier samplings, were also examined to ensure that the abnormalities observed in *A. squalicola* were not just a local trend in the Sognefjord. One of the two specimens from the Azores had two abnormally shaped cirri: one was uniramous and the other was triramous. The specimen from the Raunefjord had one uniramous, one triramous, and one missing cirrus.

#### Thoracic appendages of *L. anatifera* and *B. balanus*

For comparison, the thoracic appendages from five specimens of both *L. anatifera* and *B. balanus* were examined. Unlike *A. squalicola*, they possessed well-developed long, filamentary and highly setose cirri with clear segmentation. All specimens examined had six pairs of cirri, and no abnormalities were observed. Cirri I–VI of both species differ in their morphology, as the two rami (endopodite and exopodite) in certain pairs are of unequal lengths. However, left and right cirri in a pair were always symmetrical and had the same morphology in all specimens examined.

### Isotopic δ^13^C and δ^15^N analyses

**Fig. 7:**
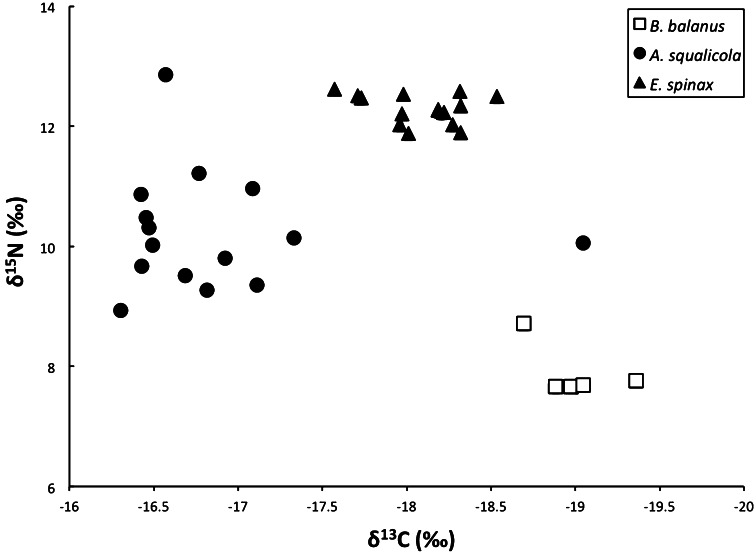
Stable isotope (δ^13^C and δ^15^N) values from *A. squalicola* (15 specimens), its host *E. spinax* (15 specimens), and the suspension-feeding barnacle *B. balanus* (5 specimens). Mean values are (δ^13^C −18.1±0.3 and (δ^15^N 12.3 ± 0.2 for *E. spinax*, δ^13^C −16.9 ± 0.7 and δ^15^N 10.2 ± 1.0 for *A. squalicola*, and δ^13^C −19.0 ± 0.2 and δ^15^N 7.9 ± 0.5 for *B. balanus*

In order to investigate the trophic level of *A. squalicola*, isotopic analyses of nitrogen and carbon were conducted from the parasite, its host (*E. spinax*) and the suspension-feeding barnacle *B. balanus* (Fig. 7).

*Balanus balanus* has the lowest values of both δ^15^N and δ^13^C. *Anelasma squalicola* has the highest values of δ^13^C, while *E. spinax* has the highest range of δ^15^N values. The average δ^15^N value of *A. squalicola* is intermediate between its host shark and the suspension-feeding barnacle.

One specimen of *A. squalicola* has δ^15^N values in the range of that of *E. spinax*, and another one has δ^13^C values in the range of *B. balanus*. Except for these two cases, there was no overlap in either the δ^15^N values or the δ^13^C values between the three species.

### Stomach contents

*Anelasma squalicola* has a complete alimentary canal. The probosciform mouth leads into a short esophagus. Following the esophagus is the stomach, as a swollen part of the alimentary canal, and the intestine as a canal following the longitudinal axis of the animal toward the anus. The canal gradually decreases in diameter from the stomach toward the posterior end and terminates in a narrow tube to the anus. The alimentary tract possesses several small folds in the stomach and the posterior part of the intestine.

All 30 dissected specimens were devoid of food items in their alimentary tracts from the mouth to the anus.

## Discussion

A recent phylogenetic study indicates that the morphologically aberrant parasitic barnacle *A. squalicola* is closely related to the pedunculate barnacle *Capitulum mitella* (Linnaeus, 1758), which is a conventional suspension-feeding barnacle restricted to the rocky intertidal zone of East-Asian shores (Rees et al. 2014). Intuitively, this relationship is surprising since *A. squalicola* is found on lantern sharks, inhabiting deep waters (Straube et al. 2010). It has, however, been estimated that the split between the linages leading to these two very different barnacle species took place about 120 million years ago (Rees et al. 2014), explaining the present differences in both ecology and morphology. While *C. mitella* remained in what is believed to be the original ecological niche of barnacle evolution, the intertidal rocky shore (Lin et al. 2015, and morphologically changed little from the common ancestor (phylogenetic niche conservatism), *A. squalicola* followed an alternative evolutionary path. At a certain point in the evolutionary past, an ancestor of *A. squalicola* must have initiated phoresy on a shark (Rees et al. 2014). Phoresy, where one organism uses another as a means of transportation, is often seen as a common step toward parasitism (Poulin 2007). Phoresy is frequent among barnacles that tend to use marine mammals, reptiles, and invertebrates, but not fishes, as their vehicles. Uniquely within Cirripedia, *A. squalicola* has successfully adapted to an existence on lantern sharks and, in contrast of being a commensal hitchhiker like all other barnacles that uses vertebrates as mobile substrates, it has developed the peduncle as a feeding device, enabling it to feed directly on the host. But although this *de novo* developed feeding mode makes a new resource available, *A. squalicola* still possesses a reduced version of the original filter feeding apparatus. Whether functional or not, the presence of the original feeding structure for suspension feeding, alongside with the embedded peduncle for parasitic nutrition uptake, indicates that *A. squalicola* represents the rare incidence of a transitional stage “caught” in a major evolutionary leap between one mode of life to a completely different one. The study of *A. squalicola* might provide an unprecedented glimpse into the macroevolutionary engine room of an organism during the brief period in which it is under strong directional selection pressure (from suspension feeding to parasitism). For an understanding of this process, the animal’s biology must be uncovered and a good place to start comes from developing a thorough understanding of its mode of feeding. In *A. squalicola* the central questions to address concerning the feeding biology are: is it still suspension feeding or has it become a facultative parasite? If so, what is happening to the non-functional filter feeding structures, vital to a conventional barnacle, but useless, or even selectively disadvantageous for a parasite? Or is *A. squalicola* utilizing a combined feeding strategy where one food source comes directly from the host, and another from planktonic organisms in the surrounding seawater via conventional suspension feeding? The most direct approach to compiling data, which at least partly could bring evidence to the topic, is a thorough examination of the stomach content of a substantial number of specimens of the barnacle.

### Stomach content

No food items were found in any of 30 examined digestive tracts of *A. squalicola*. This strongly suggests that the examined animals did not employ the alimentary tract for food processing at all. Otherwise the remains of digested food items would have been present. Although Darwin (1851) examined only one specimen, he reported that its stomach was “quite empty” but he did not elaborate any further on the topic. Johnstone and Frost (1927) also found no stomach contents in the specimens examined by them.

### Isotopic analyses

Stabile isotopes of nitrogen and carbon have been shown to indicate a stepwise enrichment with trophic level in marine systems (Hobson et al. 1994). The isotopic composition of nitrogen and carbon in marine biota can provide information concerning food sources and, therefore, also trophic levels (Wada et al. 1987). With each trophic transfer between a consumer and its diet, δ^13^C values, which are used to distinguish between primary production sources, normally increase by 1 ‰ (DeNiro and Epstein 1978), whereas δ^15^N values, which are used to determine trophic levels, normally increase by 1–6 ‰ (Minagawa and Wada 1984). At first one would expect a parasite to be enriched in both δ^13^C and δ^15^N with respect to its host, as it feeds on its host in a similar manner to a predator feeding on a prey (Pinnegar et al. 2001). However, considering the diversity of parasitic feeding modes, the picture is less simple. Endoparasites living within the digestive systems of their hosts would be expected to have similar isotopic values as their hosts, assuming they feed on the same food items as the host itself. Ectoparasites, feeding directly on host tissue, would be expected to have higher isotopic values than the host, like in a classical predator–prey relationship. These assumptions, however, are not always borne out by observations. Studies have shown that endoparasites, such as nematodes and cestodes, appear to be depleted in respect to their hosts, and ectoparasites, such as copepods and isopods have been found either enriched or depleted (Iken et al. 2001; Pinnegar et al. 2001). In regard to the host–parasite relationship between *E. spinax* and *A. squalicola*, one would initially expect *A. squalicola* to have higher isotopic values if it feeds directly on the shark via the peduncle and the original feeding appendages are non-functional. However, the mean isotopic results show that *A. squalicola* is only enriched (lower negative value) in δ^13^C, and actually slightly depleted in regard to δ^15^N compared to its host. With regard to δ^15^N, *A. squalicola* falls into the category of ectoparasites that are slightly depleted compared to their hosts. In a study on the marine food web structure using stable isotope analysis Iken et al. (2001) included a cirripede parasite from the squat lobster *Munidopsis crassa* Smith, 1885. Although the parasite was not further identified, it is likely to belong to the rhizocephalan genus *Cyphosaccus,* which is known to parasitize this host species (Lützen 1985). In their study Iken et al. (2001) reported depleted values for both δ^15^N and δ^13^C in this parasite compared to its crustacean host. Rhizocephalan barnacles infiltrate their hosts via an extensive ramifying root system, the interna, which is the only feeding organ present (Noever et al. 2015). All other structures, such as the alimentary canal or a mouth are absent, entailing that rhizocephalans exclusively have the host as the nutritional source. The similarity in the δ^15^N and δ^13^C pattern found in the indisputable parasite and *A. squalicola* indicates that the latter, like the rhizocephalan, exclusively lives off its host.

It is well documented that isotopic results, with regard to both the δ^15^N and the δ^13^C values, can vary between different organs within the same individual due to tissue specific differences in biochemical composition (Pinnegar and Polunin 1999). The slightly lower δ^15^N values found in *A. squalicola* compared to its host might be explained by the fact that muscle tissue were used for the isotope analyses of *E. spinax,* while the most likely food resource of *A. squalicola* is the interstitial fluid of the host. Both the δ^15^N and δ^13^C values of the conventional suspension-feeding barnacle *B. balanus* are substantially lower than those in *E. spinax* and *A. squalicola*, placing *B. balanus*, as a suspension feeder, at a lower trophic level than *A. squalicola.*

### Mouthpart terminology

Johnstone and Frost (1927), who conducted the previously most comprehensive morphological study on *A. squalicola*, stated that the mouth appendages consisted of a labrum, the mandibular palps, mandibles, maxillae and a “labium”. They also remarked that Darwin mentioned a pair of “outer maxillae”, but that they had not been successful in finding these. Johnstone and Frost were obviously uncertain about the organization and terminology of the mouth appendages, but suggested that the “labium” could be built up of what Darwin called the “outer maxillae”. A “labium” is, in fact, a term used to describe the lower lip of other arthropods such as insects, but it is not traditionally used to describe crustacean mouth appendages. Despite the use of a wrong terminology, it is obvious that Johnstone and Frost were referring to a “lower lip”. Darwin stated that the “outer maxillae” are united at the tips with the membrane forming the supra-esophageal hollow and that there is no doubt that the two small “outer maxillae” serve as a lower lip. His observations therefore partially correspond with those made in this study, in that the “outer maxillae” appear like two min and blunt appendages. Obviously, what Darwin referred to as the “outer maxillae”, and Johnstone and Frost as the “labium”, is in modern crustacean terminology the maxillae or second maxillae (M2 in Fig. 2). Likewise, what Darwin, and Johnstone and Frost referred to as the “maxillae”, are now termed as the maxillules or first maxillae (M1 in Fig. 2).

### The mouth and mandibles

The mouth appendages of *A. squalicola* are reduced in size. The mandibles show no consistent pattern regarding the tooth-covered distal edge and are lacking the usual setation. All examined mandibles of *A. squalicola* are asymmetrical, while of the conventional barnacle specimens, only one *L. anatifera* specimen had a slightly abnormal pattern. The setose nature of balanoid mouth appendages are functionally correlated with their capacity for suspension feeding (Anderson 1981), indicating that the mouth parts of *A. squalicola,* lacking denticles and setation, most likely are not functional as a filtering device.

For species identification, taxonomists are inclined to use morphological traits with small intraspecific and large, or at least consistent, interspecific variation. This ensures reliable separation of distinct, but otherwise morphologically similar, species. The chosen trait is often a functional, morphological character, which due to its crucial importance for the organism, is under strong stabilizing selection. This is particularly true for the mouthparts of thoracican barnacles, which have been used as a significant suite of species identification characters. The characters connected to the mandibles have been considered especially important (Darwin 1851). In the present study we have investigated the mandible variation of seven *A. squalicola* specimens and compared those with the variation found within the lepadomorph barnacle *L*. *anatifera* and the balanomorph barnacle *B*. *balanus*. Our working hypothesis was that if the mandibles of the three barnacle species were under stabilizing selection it would be reflected in low morphological diversity, due to selection against characters deviating from the morphology providing optimal functionality.

The mandibles of all seven examined specimens of *A. squalicola* showed large individual variation in contrast to those of *L. anatifera* and *B. balanus*, where hardly any intraspecific variation was detectable, even in individuals with large size variation (Fig. 5). In addition we observed strong left–right side mandible asymmetries in all studied *A. squalicola* specimens, again in contrast to the two conventional barnacle species where strict right-left symmetry was retained. This result strongly indicates that the stabilizing selection for mandible functionality that prevails in suspension-feeding barnacle species seems to have been suspended in *A. squalicola*, and the structure is most likely not functional and under reduction.

### Thoracic appendages

As with as the mouth appendages of *A. squalicola*, the thoracic appendages are also obviously reduced in size compared to a conventional barnacle. In addition to the absence of setae, this indicates that *A. squalicola* is not able to filter the ambient seawater for food items. With their long, fan-shaped and setose cirri, suspension-feeding barnacles like *L. anatifera* and *B. balanus*, in contrast, are perfectly suited for suspension feeding. Lepadomorph barnacles feed by extending their cirri and performing rhythmic beating actions, creating a current of water within the mantle for the mouth appendages to filter (Anderson 1980). Balanoid barnacles feed in a similar way, also with complex cirral rhythmic beating activities (Crisp and Southward 1961).

Not only does *A. squalicola* possess cirri with a rudimentary appearance and absence of setation, but we also observed an extremely high prevalence of specimens with abnormal cirri morphology. The usual invariable cirri symmetry of other barnacle species is no longer maintained in *A. squalicola*, resulting in remarkable individual variations. In addition to the high incidence of abnormally shaped cirri, even the number of cirri pairs in some specimens is reduced (Fig. 6). Broch (1924) reported a single cirri abnormality, but the extent of this pattern was completely unknown prior to this study. The fact that abnormal cirri are found in specimens from different locations and host species further indicates that high frequencies in asymmetries are representative for the species, and not just representation of a local trend (Table 2).

**Table 2:** Percentage of specimens of *A. squalicola* with different categories of abnormalities

Abnormality	**%**
*Uniramous cirrus*	42 specimen—75 %
*Triramous cirrus*	5 specimen—8.9 %
*Tetraramous cirrus*	3 specimen—5.4 %
*Very unequal branches*	9 specimen—16 %
*Lacking 2 different cirri*	1 specimen—1.8 %
*Lacking pair of cirri*	5 specimen—8.9 %
*Lacking cirrus*	10 specimen—17.9 %
Other	6 specimen—10.7 %

The numbers are based on a total of 56 individuals with abnormal appendages

### Comparison with *Rhizolepas*

Except for *A. squalicola* the only other thoracican barnacle species that apparently feed via the peduncle are in the polychaete-infesting genus *Rhizolepas* (Day 1939; Zevina 1968). *Rhizolepas* lacks both a mouth and an anus, but the alimentary canal is still present (Zevina 1968). Although *Rhizolepas* spp. appear to be further adapted toward parasitism than *A. squalicola,* with a profusely branching trophic organ inside its host, more similar to the interna of the Rhizocephala (Bresciani and Høeg 2001), there are some remarkable similarities between these two genera. The cirri in *Rhizolepas* spp. are consistently uniramous. As mentioned above, uniramous cirri were found in 42 % of *A. squalicola*, including one specimen with nine uniramous pairs. *Rhizolepas* spp. has the first five pairs of cirri placed anterior to the penis, while the sixth pair projects dorsally from it (Day 1939). With this peculiar, apparently fixed morphological feature of *Rhizolepas* spp., it is interesting that a sixth cirrus was found posteriorly to the penis in one specimen of *A. squalicola* in our study. This specimen had, in addition to the *Rhizolepas*-like location of the penis, two further penises at the same site (Fig. 6c).

### The penis of *A. squalicola*

The occurrence of specimens with multiple penises was not an uncommon abnormality of *A. squalicola* in our study. In addition, in all examined *A. squalicola* specimens, the penises were found to be very short. Due to their sedentary life style, thoracican barnacles usually possess exceptionally long, extendable penises, enabling them to reach and inject sperm into the mantle cavity of a neighboring conspecific, despite an inability to reposition from the original attachment site. The small penis size for a thoracican barnacle found in *A. squalicola*, in addition to high frequency of duplications of this crucial mating structure, might indicate that reproduction follows a different pattern than in other barnacle species.

## Concluding remarks

Based on the findings of our study, comprising stable isotope data, investigation of the alimentary tract, as well as mouth parts and cirri comparisons of *A. squalicola* and two suspension-feeding barnacle species, we conclude that *A. squalicola* is incapable of conventional suspension feeding and is a facultative parasite. *Anelasma squalicola* is using the functionally and morphological unrelated host-embedded peduncle as the sole feeding device (Fig. 1c, d). Beside its globular shape, ensuring solid anchorage in the tissue of the host, the rootlets enlarge the peduncle’s surface area considerably, which must be expected of a device believed to absorb nutrients from the shark and to be the barnacle’s sole functional feeding structure. Morphological structures critical to the survival of conventional barnacles, such as the mouthparts, cirri, and most likely also the alimentary tract, have lost their functionality in *A. squalicola*.

Assuming that it is energy demanding to maintain structures without function, there must be a selection reward for reducing them. Following this reasoning, the selection pressure for optimizing the structures that support the novel parasitic feeding mode, peduncle feeding, must be considerable and the evolutionary window where traces of both feeding systems simultaneously are present is likely to be ephemeral.

Our study provides a rare insight into the fate of previously vital morphological structures in an organism, which recently have lost their function. For the feeding apparatus of *A. squalicola*, this can be summarized as to size-reduction, morphological simplification, increased individual variation, high frequencies of morphological anomalies, and a high instance of left–right asymmetries.
